# A novel prognostic signature in osteosarcoma characterised from the perspective of unfolded protein response

**DOI:** 10.1002/ctm2.750

**Published:** 2022-03-28

**Authors:** Chengcheng Shi, Faming Zhao, Tingting Zhang, Denghui Xu, Zhuangyu Hao, Fengzhen Cui, Ji‐Hua Shi, Yang Jin, Ningning Li, Caihong Yang, Yi Zhang, Xia Sheng

**Affiliations:** ^1^ Department of Pharmacy The First Affiliated Hospital of Zhengzhou University Zhengzhou China; ^2^ Key Laboratory of Environmental Health Ministry of Education & Ministry of Environmental Protection School of Public Health Tongji Medical College Huazhong University of Science and Technology Wuhan China; ^3^ Department of Orthopaedic Surgery The First Affiliated Hospital of Zhengzhou University Zhengzhou China; ^4^ Department of Hepatobiliary and Pancreatic Surgery Henan Key Laboratory of Digestive Organ transplantation The First Affiliated Hospital of Zhengzhou University, Zhengzhou University Zhengzhou China; ^5^ The Seventh Affiliated Hospital of Sun Yat‐sen University Shenzhen China; ^6^ Department of Orthopedics Tongji Hospital Tongji Medical College Huazhong University of Science and Technology Wuhan China

Dear Editor,

Osteosarcoma (OS) is the most common primary malignant tumour of bone with variable molecular biology and prognosis. This makes better patient stratification and precision treatment an urgent clinical need.^1^ Activation of the unfolded protein response (UPR) is a hallmark of cancer cells facing endoplasmic reticulum (ER) stress,^2^, ^3^ yet its clinical relevance in OS remains to be explored. By comprehensive interrogation of OS datasets established by us and others,^4^, ^5^, ^6^, ^7^ the present study consolidates UPR activation as a critical molecular feature of OS and refines a prognostic gene signature from this perspective with translational potential.

In this study (see Figure S1A for workflow), we assembled 5 independent OS cohorts (GSE99671, GSE126209, GSE21257, TARGET and Zhengzhou datasets), plus the TCGA sarcoma dataset. Three datasets (GSE99671, GSE126209 and Zhengzhou) with paired tumour and normal tissues were analysed for deregulated genes; two of them with relatively large sample size were further selected for pathway enrichment. Three datasets (GSE21257, TARGET and TCGA) with solely tumours and survival information for patient classification and prognostic model construction (Table S1).

We first interrogated GSE99671 with paired tumour and normal samples, and identified 1581 differentially expressed genes (DEGs) [|log2 (fold change)| > .5 and adjust *p* value < .05] (Figure S1B). To our interest, several pathways related to ER function, such as response to ER stress and protein processing in ER, ranked top according to Gene Ontology (GO) and Kyoto Encyclopedia of Genes and Genomes (KEGG) analyses (Figure 1A and B). Enrichment of UPR and MYC targets was verified by Hallmark Gene Set Enrichement Analysis (GSEA) (Figures 1C and S1C), mirroring the recently established co‐activation of UPR and MYC in multiple cancers.^4^, ^8^

**FIGURE 1:**
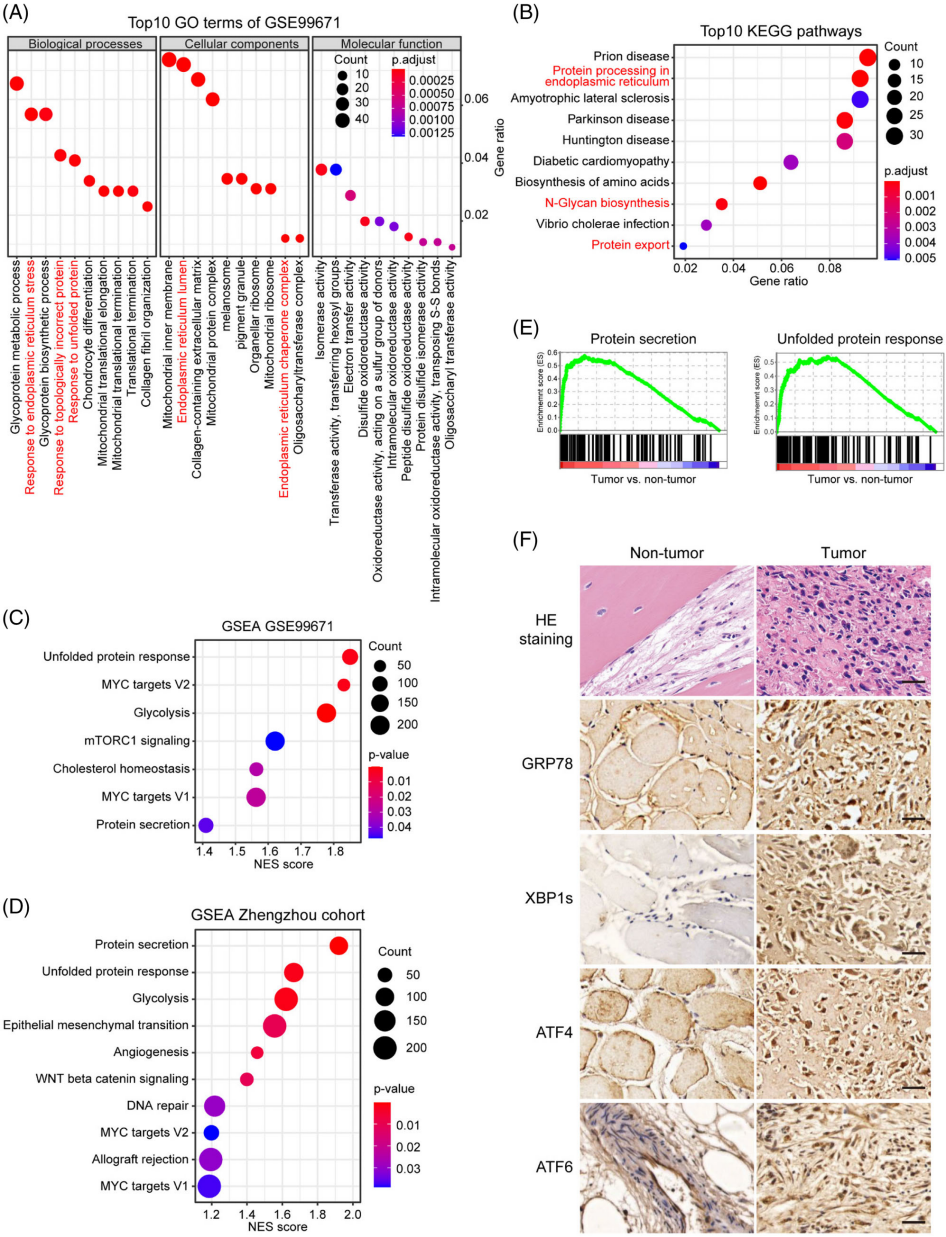
UPR is activated in multiple human OS cohorts. (A), (B) Top 10 most enriched GO terms (A) and KEGG pathways (B) of the upregulated DEGs. (C), (D) GSEA analyses of the GSE99671 (C) and Zhengzhou (D) cohorts. (E) GSEA plot of the Hallmark protein secretion and UPR pathways. (F) Representative images of hematoxylin and eosin (HE) and immunohistochemical staining of GRP78, XBP1s, ATF4, and ATF6 in OS foci and matched normal tissues from the Zhengzhou cohort. Scale bars, 100 μm

Meanwhile, transcriptomic analyses on 24 matched tumour and normal tissues collected in our hospital (hereafter referred to as Zhengzhou cohort) enriched similar Hallmark pathways such as protein secretion, UPR and MYC signalling (Figure 1D and E). Consistently, immunohistochemistry observed markedly elevated level of GRP78, and nuclear localisation of canonical UPR transcription factors XBP1s, ATF4, and ATF6 in tumour foci compared to normal tissues (Figure 1F). By overlapping a repertoire of previously described UPR‐related genes^4^, ^5^, ^6^, ^7^ (Table S2) with DEGs in the GSE99671, GSE126209 and Zhengzhou cohorts, we acquired 14 genes with significantly aberrant expression in OS (Figures S1D and S2A), defined as the OS‐specific UPR gene signature.

Based on this signature, we constructed a set of scoring system^9^ to quantify the UPR activity of each tumour (termed as UPR score) and conducted unsupervised consensus clustering^10^ to classify different molecular features and prognosis. Interestingly, patients from GSE21257 were clustered into two subtypes with notable difference in global gene expression, such as genesets related to ER biology and UPR, as well as in overall and progression‐free survival (Figures 2 and S2B–E). Likewise, this protocol led to optimal bifurcation of patients in both the TARGET OS and TCGA sarcoma datasets (Figures 2C and D and S2F and G).

**FIGURE 2:**
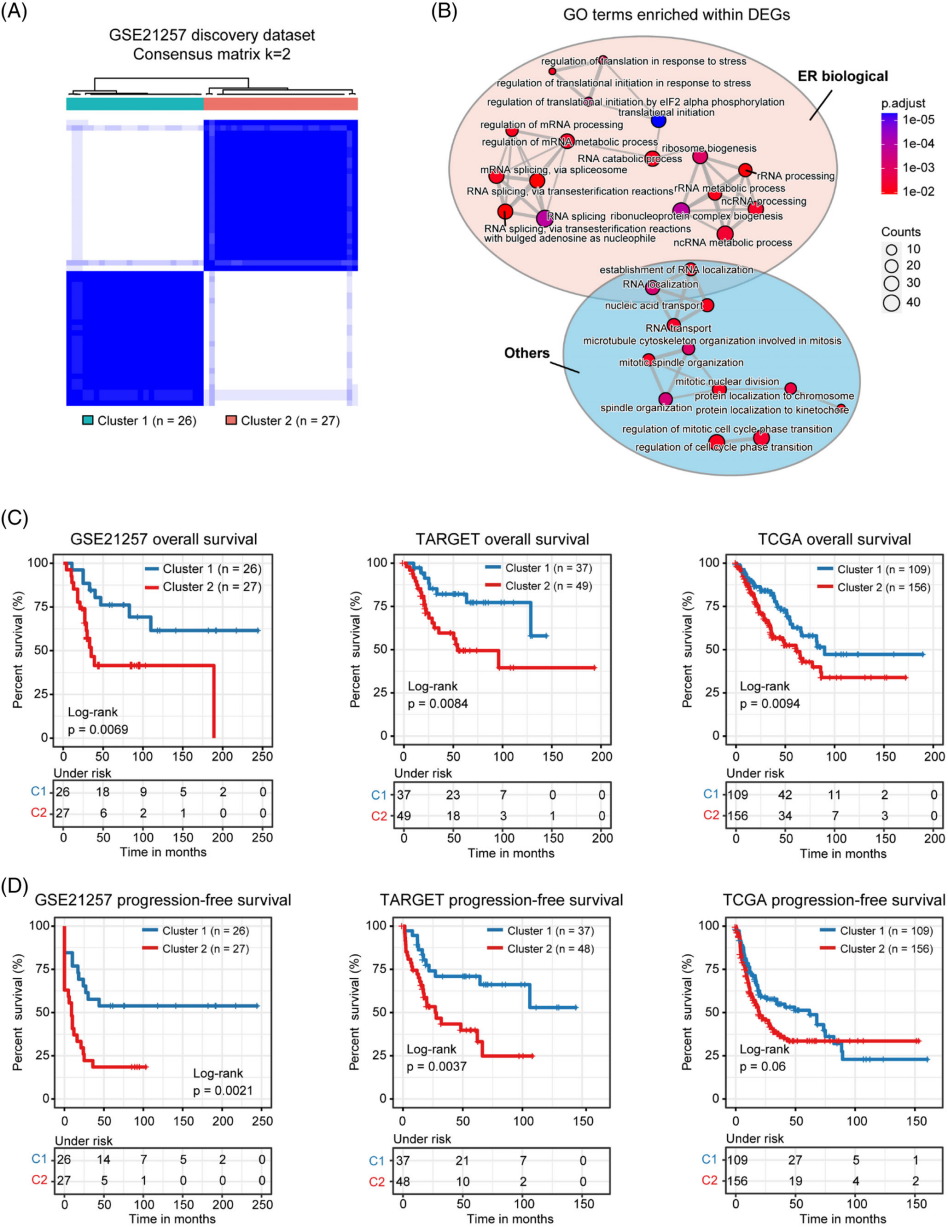
Classification of two molecular subtypes of OS patients. (A) Consensus matrix heatmaps (*k* = 2) of UPR gene signature in GSE21257 cohort. (B) The bubble pattern displays the ER function‐related biological processes significantly enriched in GSE21257 cohort. (C), (D) Kaplan–Meier curves of overall survival (C) and progression‐free survival (D) for the two clusters

From a translational standpoint, we were further interested in developing a prognostic signature consisting of a handful of genes with higher power. Based on the classification strategy above, we applied Kaplan–Meier survival analyses coupled with univariate Cox regression to GSE21257 and TARGET cohorts and identified 21 candidate DEGs (Table S3). Using GSE21257 as discovery dataset, LASSO Cox regression based on overall survival and patient status established a linear model as follows: risk score = 2^ (0.4528 × expression level of NOP58 − 0.2303 × expression level of ALOX5AP + 0.2209 × expression level of MYC + 0.0828 × expression level of LGR4 + 0.0209 × expression level of GADD45GIP1) (Figure S3A). Subsequent survival analyses confirmed that the overall and progression‐free survival of the high‐risk subgroup was significantly shorter than the low‐risk subgroup (Figure 3A). Receiver Operator Characteristic (ROC) curve analyses indicated that the 1‐, 6‐, and 12‐year area under curve (AUC) values were .83, .83 and .82, respectively, for overall survival, while .83, .80 and .86 for progression‐free survival (Figure S3B). Of these five genes, the expression of NOP58, MYC, LGR4, and GADD45GIP1 was significantly higher in the high‐risk subgroup, whereas that of ALOX5AP was conversely profiled (Figure 3B). Importantly, the level of NOP58 and ALOX5AP, and their correlation with OS pathology was validated in independent tissue microarrays (Figure 3C–E; Table S4). Subsequent analyses of TARGET OS and TCGA sarcoma cohorts as validation datasets similarly subtyped patients with distinct status of risk scores, UPR activity, molecular charateristics and survival outcomes (Figures S3C–G and S4A).

**FIGURE 3:**
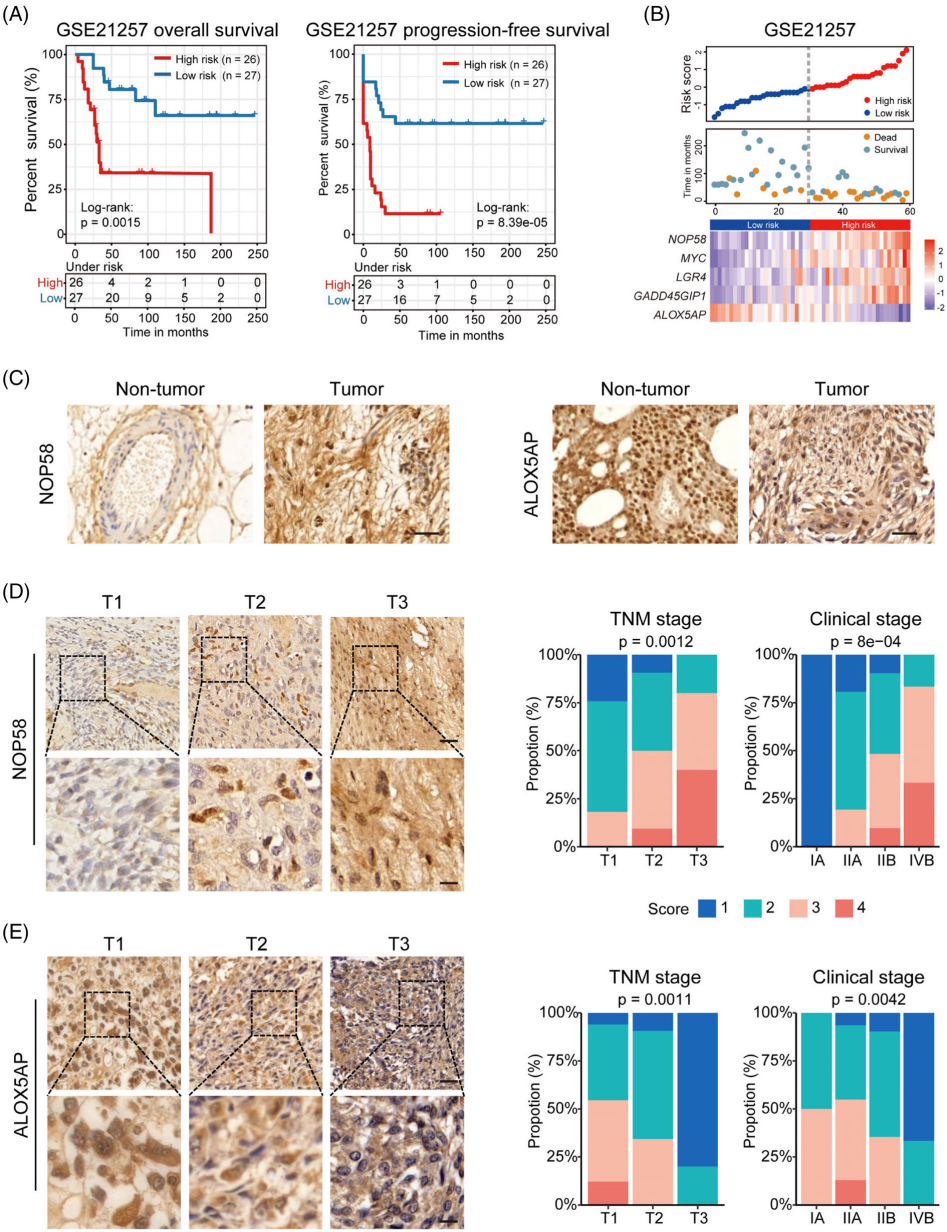
Identification and validation of the OS prognostic gene signature. (A) Kaplan–Meier curves for overall (left) and progression‐free (right) survival divided by risk score in the GSE21257 discovery set. (B) Distribution of risk scores and survival status, as well as expression profile of the five‐gene panel in the GSE21257 cohort. (C) Representative immunohistochemical images of NOP58 (left) or ALOX5AP (right) in OS foci and normal tissues from the Zhengzhou cohort. Scale bars, 100 μm. (D) NOP58 was stained and scored in an OS tissue microarray consisting of 70 tumours. Representative images in tumours of different TNM stages (left) and distribution of NOP58 score across different TNM and clinical stages (right) are shown. (E) Similar analyses was conducted for ALOX5AP. Scale bars, 100 μm (upper panel) and 30 μm (lower panel). The Kruskal–Wallis test by ranks

Additionally, we observed significant enrichment of multiple immune‐relevant gene signatures according to pathway enrichment analyses (Figures 4A and S4B–D). In fact, all the high‐risk subgroups across different datasets uniformly showed lower stromal and immune scores, but higher tumour purity and stemness compared to the low‐risk subgroups (Figures 4B and S5A–D). Dissection of immune infiltration by CIBERSORT uncovered significantly higher proportion of CD8^+^ T cells, monocytes and M2 macrophages, and lower proportion of memory resting CD4^+^ T cells and M0 macrophages in the low‐risk subgroup (Figures 4C and S5E and F). Interestingly, the level of several immune checkpoints was markedly higher in the low‐risk, including PD‐L2, CD86, TNFRSF14, CD4 and LAG3 (Figures 4D and S6A–C). Submap analyses confirmed that the low‐risk subtype was more likely to respond to anti‐PD1 therapy (Figures 4E and S6D and E), which warrants future investigation.

**FIGURE 4:**
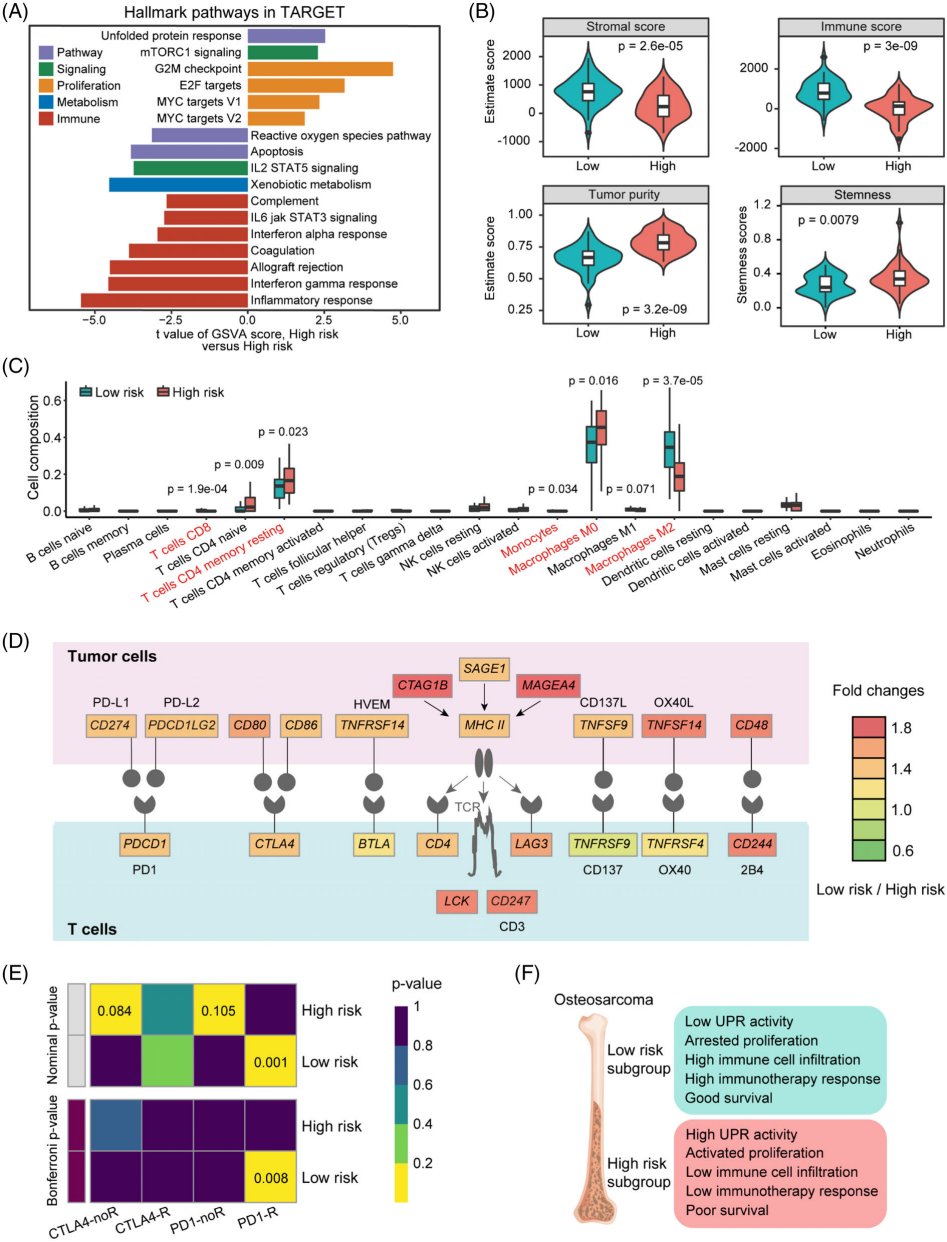
Immunological landscape of the two risk subgroups. (A) Differences in pathway activities scored per sample by GSVA between high‐risk and low‐risk subgroups. Shown are *t* values from a linear model. V1, version 1; V2, version 2. (B) The distribution of stromal score, immune score, tumour purity, and stemness score between the two risk subgroups in the TARGET cohort. (C) Subgroup comparison of infiltrating levels of 22 immune cell types in the TARGET cohort. Wilcoxon's rank‐sum tests. (D) Diagram of the different activities of the immune checkpoint pathway between the two risk subgroups. (E) Sensitivity prediction of different subgroups to the two immune checkpoint inhibitors in the TARGET cohort. No response, noR; response, R. (F) Schematic summary of molecular and prognostic features for subtypes categorised in this study

In conclusion, our study underlines that UPR activation is a common molecular feature of OS, and offers a novel prognostic gene signature refined from this perspective with translational value (Figure 4F).

## CONFLICT OF INTEREST

The authors declare no conflict of interest.

## Supporting information

SUPPORTING INFORMATION

SUPPORTING INFORMATION
