# Supplementary material for: A novel prognostic signature in osteosarcoma characterised from the perspective of unfolded protein response
Source: Clin Transl Med. 2022 Mar 28;12(3):e750. doi: 10.1002/ctm2.750 (PMC8958346; doi:10.1002/ctm2.750)
Supplement: Supplementary file 1 — SUPPORTING INFORMATION [file CTM2-12-e750-s001.docx]

**Supplementary information**

**A novel prognostic signature in osteosarcoma characterized from the perspective of unfolded protein response**

Chengcheng Shi^#^, Faming Zhao^#^, Tingting Zhang^#^, Denghui Xu, Zhuangyu Hao, Fengzhen Cui, Ji-Hua Shi, Yang Jin, Ningning Li, Caihong Yang, Yi Zhang*, Xia Sheng*

*Corresponding author. Email: zhangyi@zzu.edu.cn (Y.Z.); xiasheng@hust.edu.cn (X.S.).

**Table of content**

1. **Supplementary Figures**

Figure S1. UPR is activated in the both public and Zhengzhou cohorts.

Figure S2. UPR gene signature classify two molecular subtypes in public datasets.

Figure S3. Validation of the prognostic gene signature.

Figure S4. Molecular characteristics of the two risk subgroups.

Figure S5. Immunological characterization of two risk subgroups.

Figure S6. Prediction of immunotherapy responsiveness of different subgroups.

1. **Supplementary Tables (See Excel files)**

Supplementary Table 1. List of independent cohorts used in this study.

Supplementary Table 2. The previously established UPR related genes.

Supplementary Table 3. The common candidate prognostic genes in the GSE21257 and TARGET cohort.

Supplementary Table 4. Detailed information of TMA.

**Supplementary Figures**

**Supplementary Figure 1**

**
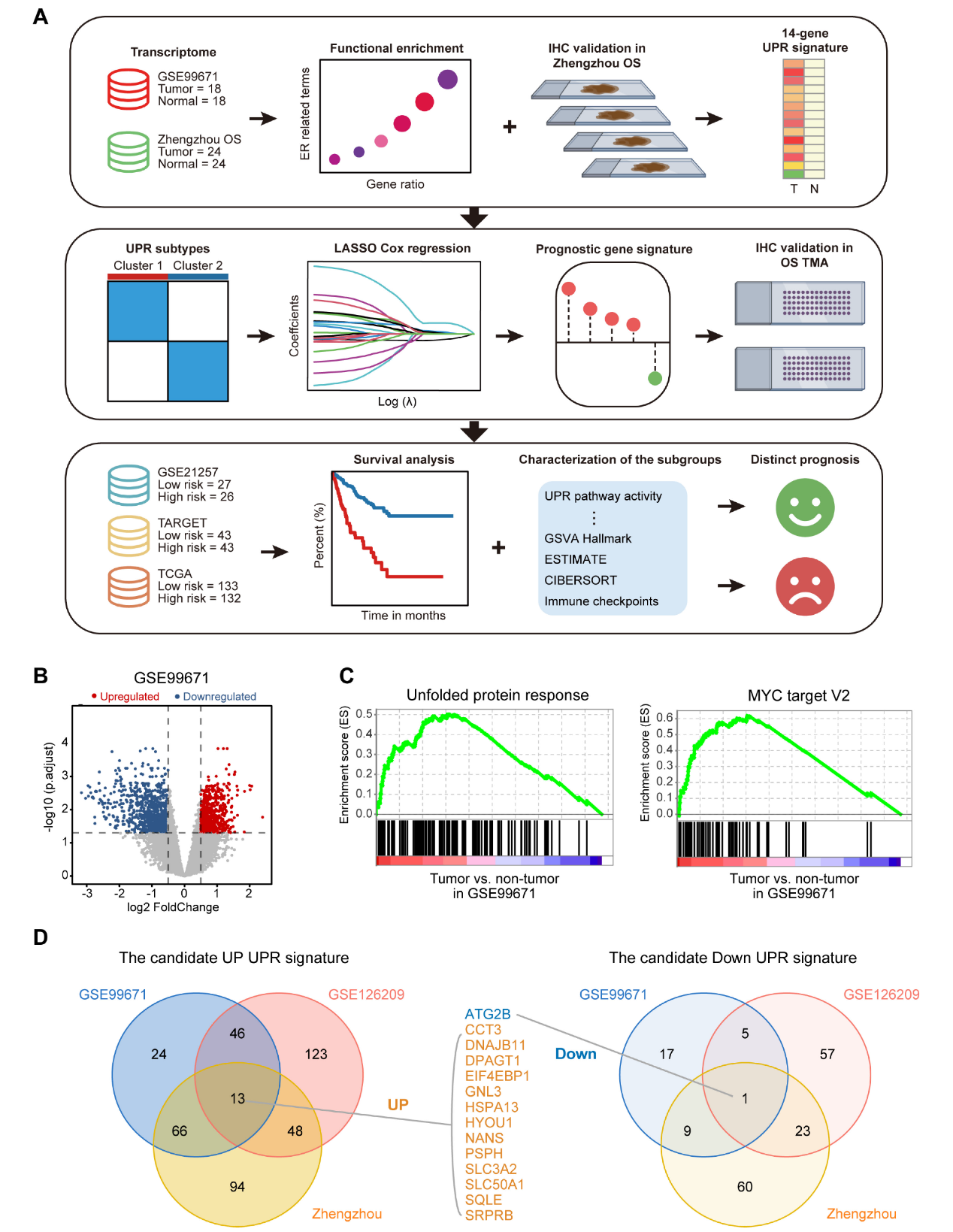
**

**Figure S1. UPR is activated in the both public and Zhengzhou cohorts.**

**A.** The workflow of this study. **B.** Volcano plot represented the 1581 DEGs between tumor tissues (n = 18) and non-tumor tissues (n = 18), with the criteria of |log2 FoldChange| > 0.5, adjusted p-value (p.adjust) < 0.05. Red, 705 upregulated DEGs; blue, 876 downregulated DEGs. **C.** GSEA plot of the Hallmark UPR and Myc targets v2 pathways. **D.** Venn diagram of differentially expressed UPR gene signatures in GSE99671, GSE126209, and Zhengzhou datasets.

**Supplementary Figure 2**

**
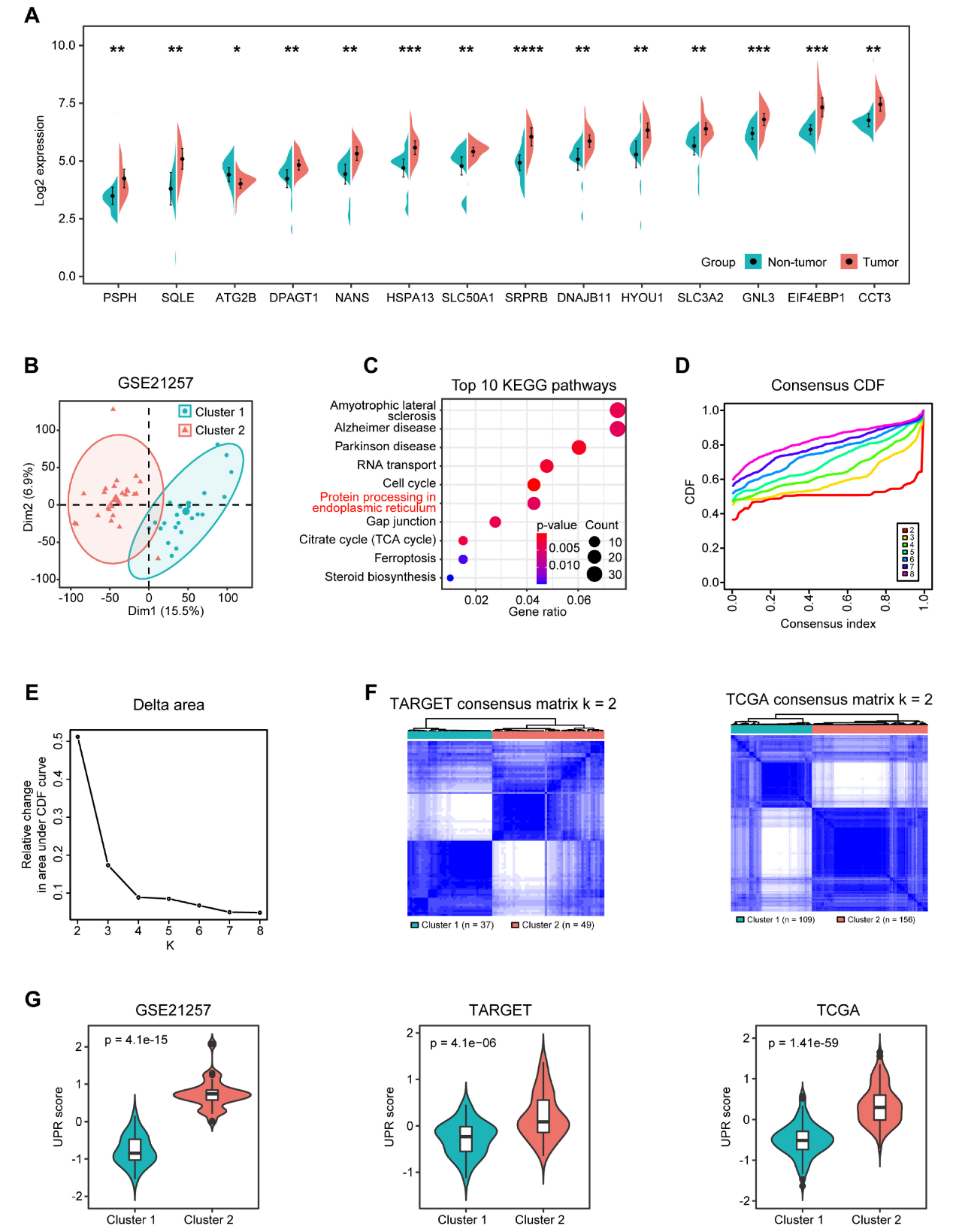
**

**Figure S2. UPR gene signature classify two molecular subtypes in public datasets.**

**A.** Expression box plot for 14 OS-specific UPR signature genes in the Zhengzhou cohort. Student’s t-test, * p < 0.05; ** p < 0.01; *** p <0.001. **B.** Principle component analysis plot of the GSE21257 cohort. **C.** The bubble pattern showed the KEGG pathways significantly enriched from the upregulated DEGs in GSE21257. **D and E.** Consensus clustering cumulative distribution function (CDF, D) and relative change in area under the CDF curve (E) for k = 2 to 8. **F.** Identification of two UPR subtypes in TARGET or TCGA cohorts. **G.** Comparison of UPR score between the two clusters. Wilcoxon’s rank-sum tests.

**Supplementary Figure 3**

**
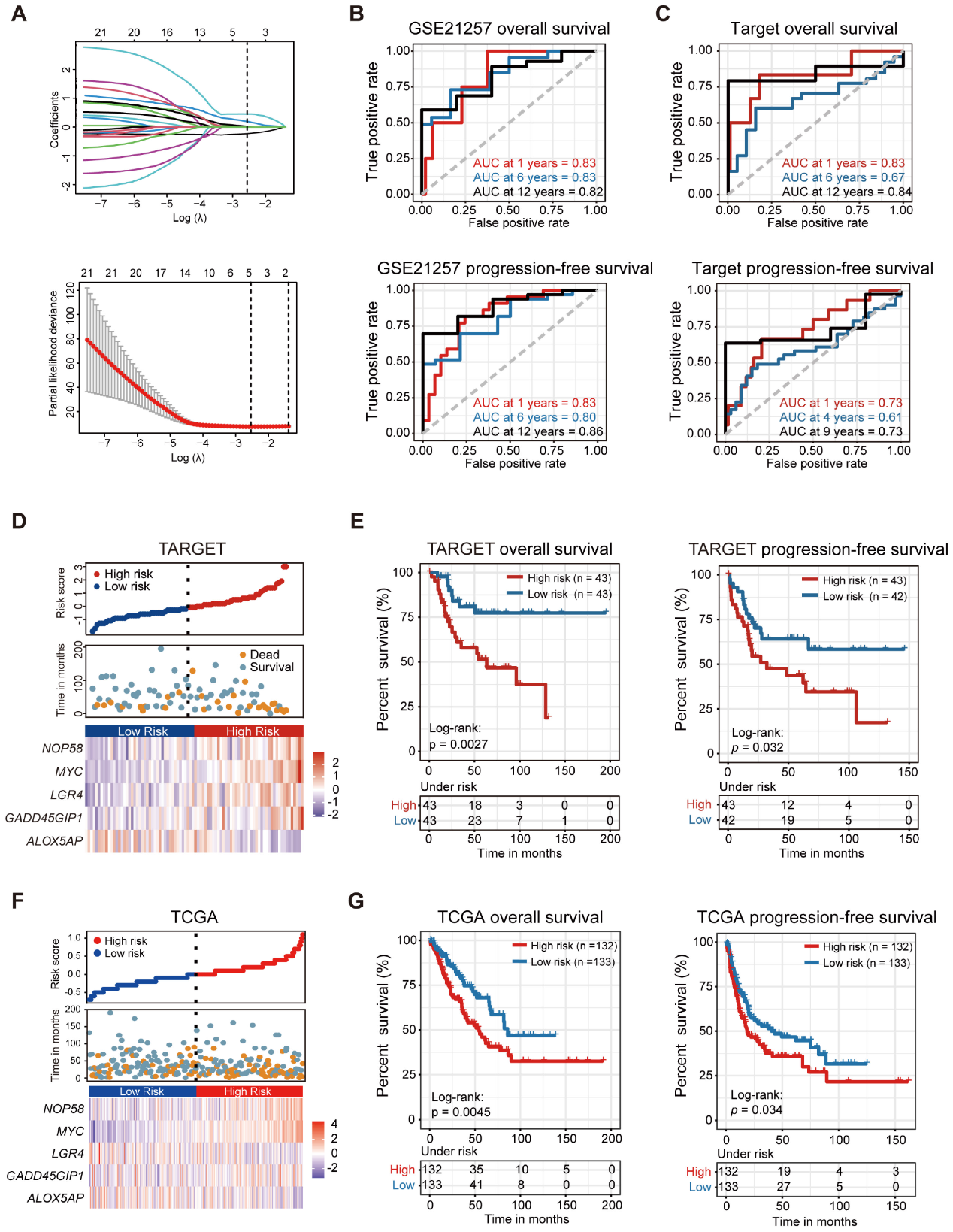
**

**Figure S3. Validation of the prognostic gene signature.**

**A.** LASSO coefficient profiles of the candidate overall survival related genes (upper panel). Optimal parameter (λ) selection by using 10-fold cross-validation (lower panel). The dotted vertical lines are drawn at the optimal values by using the minimum criteria (lambda.min, left vertical dotted line) and the one standard error of the minimum criteria (lambda.1se, right vertical dotted line). **B and C.** ROC curves of the five-gene panel for predicting the overall or progression-free survival in the two risk subgroups. **D.** Distribution of risk scores and survival status, and expression profile of the five-gene panel in the TARGET cohort. **E.** Kaplan-Meier curves of overall (left) and progression-free (right) survival based on the risk score in the TARGET cohort. **F and G.** Similar analyses was conducted for TCGA sarcoma dataset.

**Supplementary Figure 4**

**
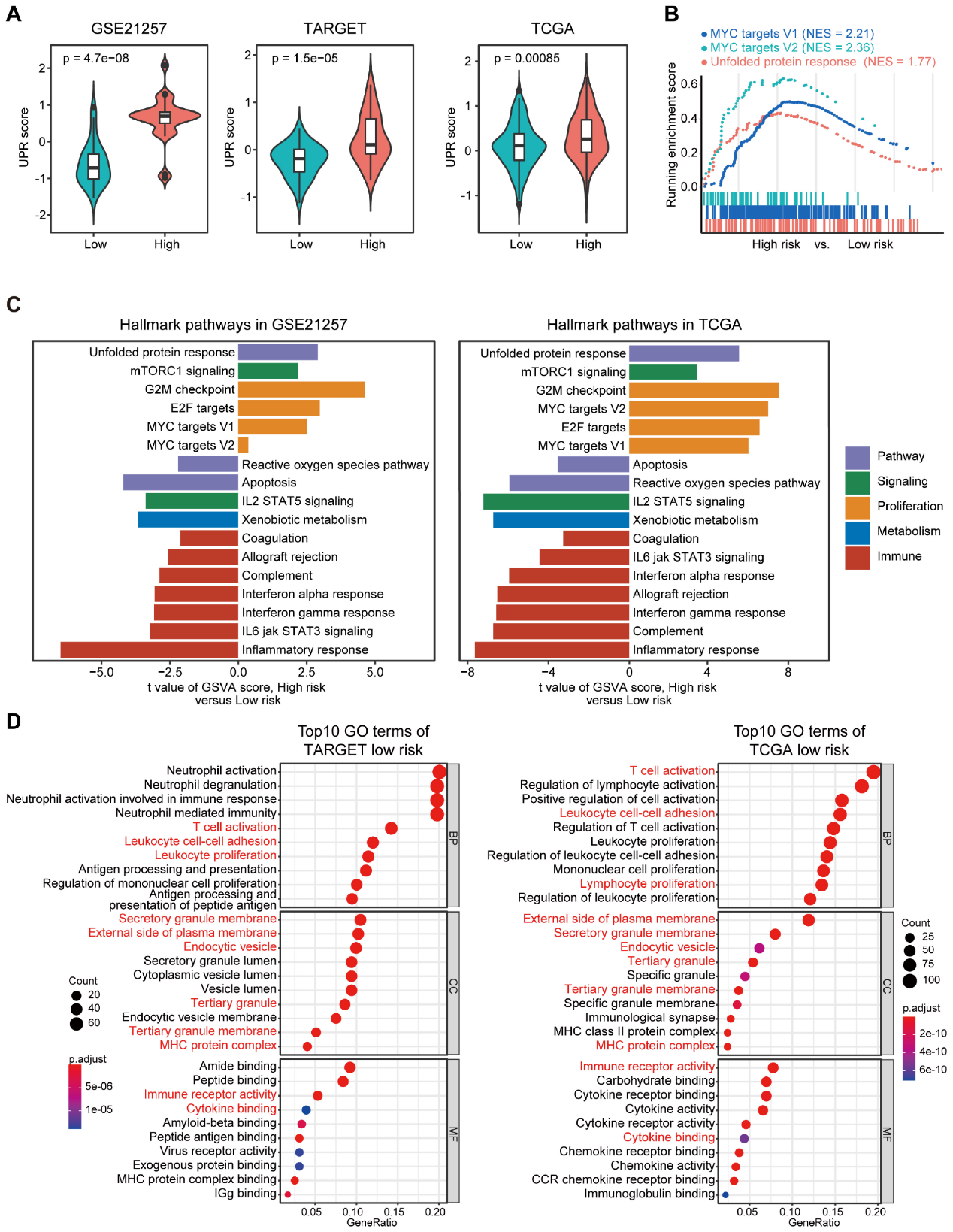
**

**Figure S4. Molecular characteristics of the two risk subgroups.**

**A.** The distribution of UPR score for the two risk subgroups. **B.** Representative images of the significantly enriched pathways. **C.** Differences in pathway activities scored per sample by GSVA between high-risk and low-risk subgroups. **D.** Top 10 most enriched GO terms of upregulated DEGs in the high-risk subgroup compared to low-risk subgroup. 392 downregulated DEGs in the TARGET cohort (left) and 565 downregulated DEGs in the TCGA (right) were analyzed.

**Supplementary Figure 5**

**
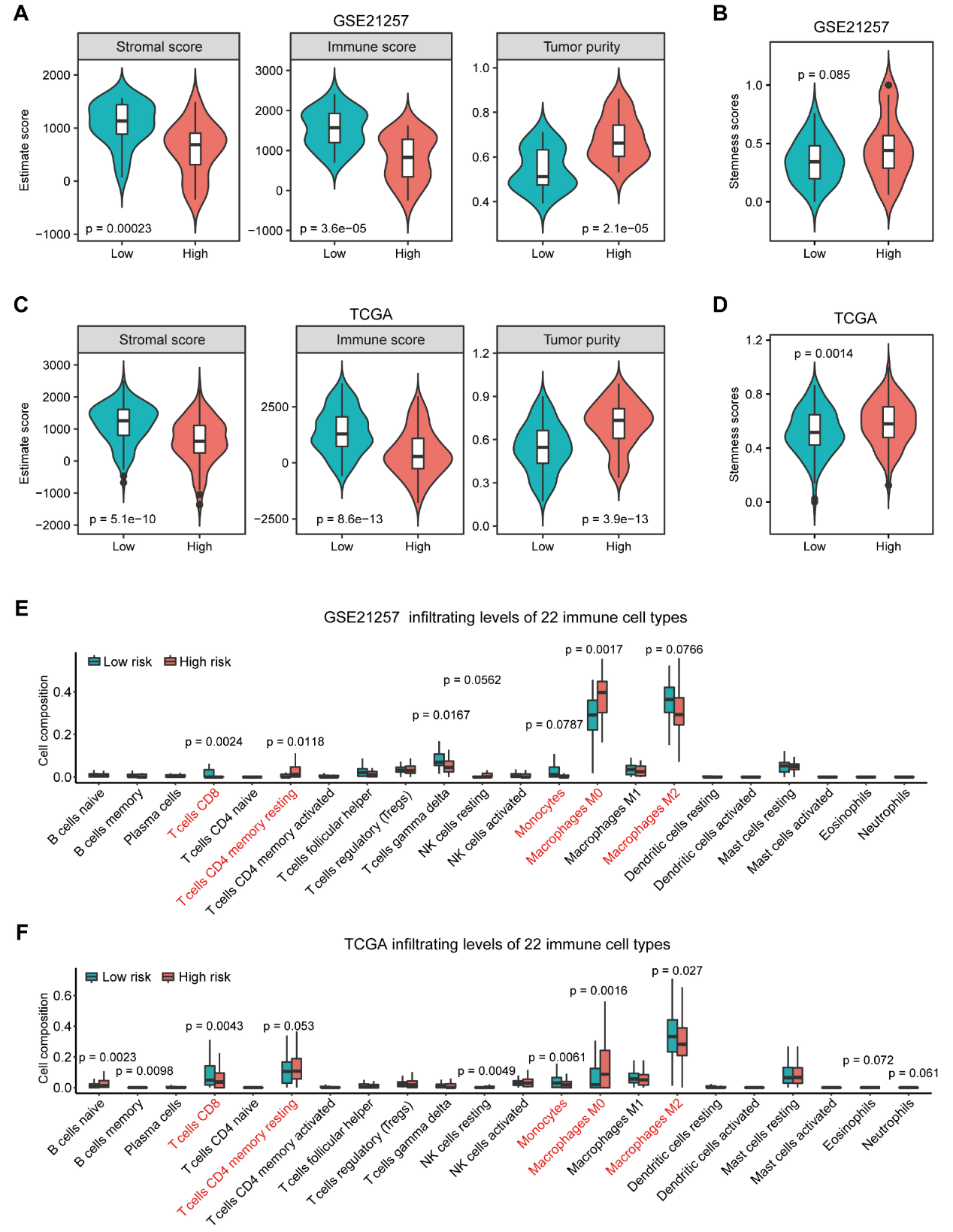
**

**Figure S5. Immunological characterization of two risk subgroups.**

**A-D.** The distribution of stromal score, immune score, tumor purity, and stemness score between the two risk subgroups in the GSE21257 (A and B) and TCGA (C and D) cohorts. **E and F.** The infiltrating levels of 22 immune cell types in two risk subgroups in the GSE21257 (E) and TCGA (F) cohorts.

**Supplementary Figure 6**


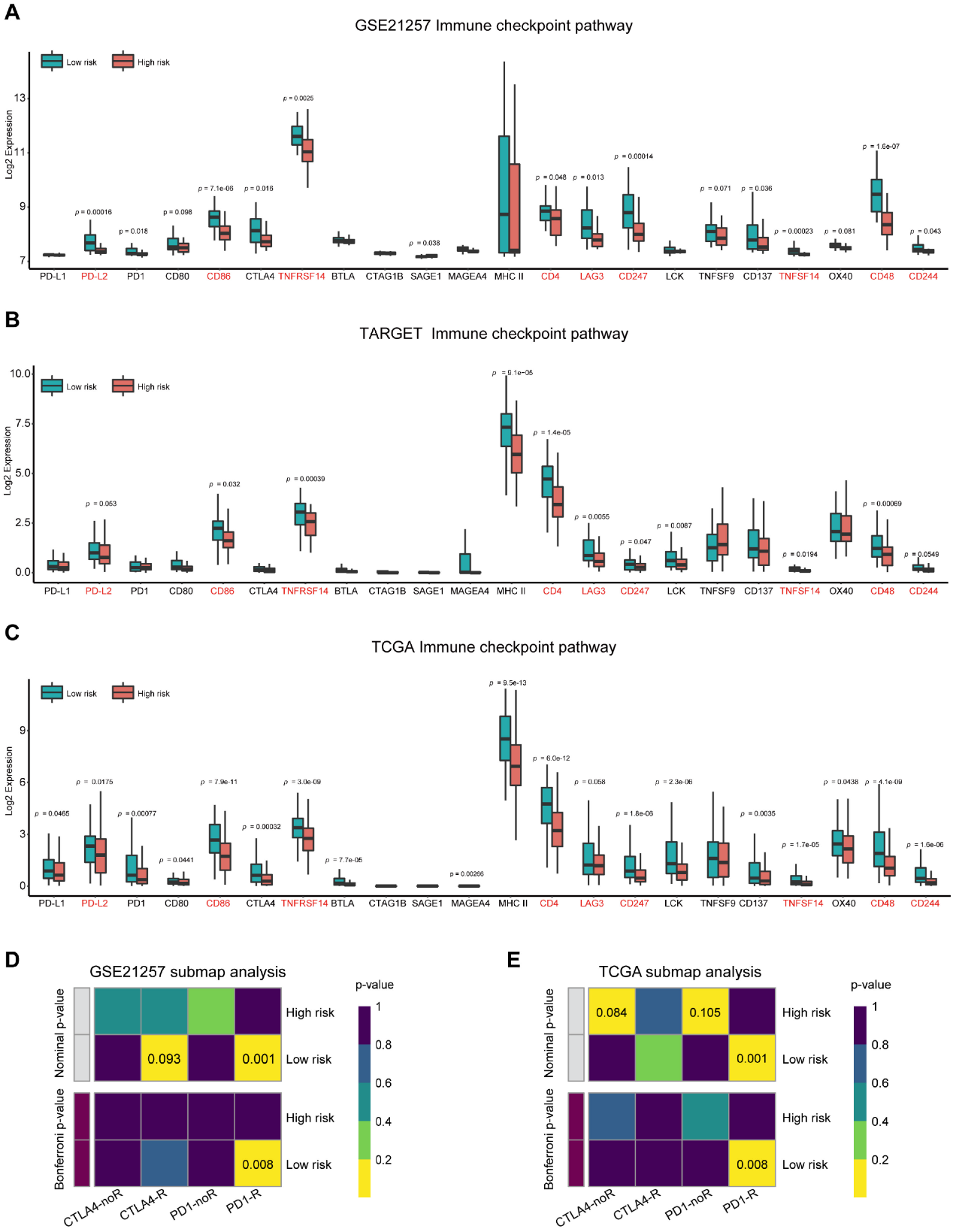


**Figure S6. Prediction of immunotherapy responsiveness of different subgroups.**

**A-C.** The expression of immune checkpoint pathway for different subgroups in GSE21257 (A), TARGET (B) and TCGA (C) cohorts. **D and E.** Sensitivity prediction of different subgroups to immune checkpoint inhibitors in GSE21257 (D) and TCGA (E) cohorts. No response, noR; response, R.
